# Role of RNH1 in the regulation of RNase H2 function

**DOI:** 10.1186/1546-0096-13-S1-O3

**Published:** 2015-09-28

**Authors:** B Kind, F Schmidt, S Kretschmer, A Shevchenko, MA Lee-Kirsch

**Affiliations:** 1 Medizinische Fakultät Carl Gustav Carus, Technische Universität Dresden, Department of Pediatrics, Dresden, Germany; 2 Max Planck Institute of Molecular Cell Biology and Genetics, Dresden, Germany

## 

Ribonuclease H2 plays an essential role for genome stability as it removes ribonucleotides misincorporated into genomic DNA by replicative polymerases and resolves RNA/DNA hybrids. Hypomorphic loss-of-function mutations in the genes encoding the three RNase H2 subunits cause the type I interferonopathies Aicardi-Goutières syndrome (AGS) and systemic lypus erythemotosus (SLE). We showed that in patients with AGS and SLE mutations cause enhanced levels of ribonucleotides in genomic DNA. We analyzed the proteomic environment of the RNase H2 complex and identified RNase Inhibitor 1 (RNH1) as an interactor. We validated the interaction of RNH1 with RNase H2 on an endogenous level using co-immunoprecipitation. Furthermore, we demonstrated that a siRNA-induced knockdown of RNH1 in HeLa cells causes low level DNA damage, activation of p53 and up-regulation of type I interferon-stimulated genes. These findings suggest a role of RNH1 in the regulation of RNase H2 function and implicate RNH1 in AGS pathogenesis.

